# The Dawn of the Professional Spirit

**Published:** 1921-01-08

**Authors:** 


### THE MATRONS' AND SISTERS' DEPARTMENT.

### THE DAWN OF THE PROFESSIONAL SPIRIT.

RATHER unreasonable demands are being made of nurses in general at this highly critical time. Many burning questions relating to salaries, hours on duty, unemployment, and the like have to be solved. It taxes the brains of men and women long accustomed to study all the factors in the nurse's career to form a judgment on these matters. There is much to be said for and against almost every line of action which can be suggested. None can exactly forecast what the effect of legislation will be. Yet, at a moment when wisdom calls out imperatively for caution, delay, and compromise, we are being told that these delicate matters can and ought to be solved by the rough-and-ready mode of a plebiscite. Because the heads of the profession are at a loss, and fail even in solemn conclave to reach a conclusion, we are to leave these vital questions to be settled offhand by "individual nursing opinion," regardless of the fact that very few nurses indeed have had any opportunity of understanding what the effect of their decisions will be. The individual nurse as we find her is still the creature of her own narrow environment. With many admirable exceptions, nurses as a class are prone to shut their eyes to all professional considerations which do not touch their momentary convenience, their momentary interests. They are myopic, and the problem is to fit them with glasses.

The College of Nursing has set itself the gigantic task of introducing among widely scattered units of the profession a common ground of interest. It is their part to build up a professional sense. We note the dawnings of it in many directions. It is a plant easily checked, of slow growth, but the great hope for its spread is the fact that, once set stirring in any locality, it throws off shoots and propagates of itself. Every Centre of the College is a place for the development of this priceless consciousness of common aims and common interests. Little struggling communities, thankful to secure a quorum at committees, content with but a fair sprinkling at meetings, hampered by tardy payment of subscriptions, for ever confronted with the "what is the good of it?" spirit, are yet doing work of more value to the nursing profession than they know. They are the nurseries of the professional spirit, and every time nursing questions are discussed questions are asked and answered, explanations are given, and timidity in speech is overcome, the hard sod which hinders growth is turned, and a result, infinitesimal it may be, follows.

The war has left us all in a very impatient spirit. We want to see things happen instantaneously, and we forget that, while we have grown more eager, the wheels go round just as slowly as they used to do. When our esteemed contributor "Ierne" appeals for an instant righting of all wrongs, for a clear estimate of the extent to which the opinions and ambitions of British trained nurses are being voiced and represented, we are all the better for the challenge. It is a reminder that much is expected, that the professional spirit is awake and impatient. There never was any moment in nursing history when pessimism was more out of place. To look back on the state of professional opinion five years ago is to look forward with sound confidence to the next five. It is not always wise policy to look behind the back. But there are times —perhaps this is one—when a backward glance reveals the fact that the worst bit of the road is over.

